# Maresin 1, a Proresolving Lipid Mediator, Mitigates Carbon Tetrachloride-Induced Liver Injury in Mice

**DOI:** 10.1155/2016/9203716

**Published:** 2016-01-06

**Authors:** Ruidong Li, Yaxin Wang, Ende Zhao, Ke Wu, Wei Li, Liang Shi, Di Wang, Gengchen Xie, Yuping Yin, Meizhou Deng, Peng Zhang, Kaixiong Tao

**Affiliations:** ^1^Department of General Surgery, Union Hospital, Tongji Medical College, Huazhong University of Science and Technology, No. 1277, Jiefang Avenue, Wuhan 430022, China; ^2^Department of Anesthesiology and Critical Care, Union Hospital, Tongji Medical College, Huazhong University of Science and Technology, No. 1277, Jiefang Avenue, Wuhan 430022, China; ^3^Department of Clinical Laboratory, Union Hospital, Tongji Medical College, Huazhong University of Science and Technology, No. 1277, Jiefang Avenue, Wuhan 430022, China

## Abstract

Maresin 1 (MaR 1) was recently reported to have protective properties in several different animal models of acute inflammation by inhibiting inflammatory response. However, its function in acute liver injury is still unknown. To address this question, we induced liver injury in BALB/c mice with intraperitoneal injection of carbon tetrachloride with or without treatment of MaR 1. Our data showed that MaR 1 attenuated hepatic injury, oxidative stress, and lipid peroxidation induced by carbon tetrachloride, as evidenced by increased thiobarbituric acid reactive substances and reactive oxygen species levels were inhibited by treatment of MaR 1. Furthermore, MaR 1 increased activities of antioxidative mediators in carbon tetrachloride-treated mice liver. MaR 1 decreased indices of inflammatory mediators such as tumor necrosis factor-*α*, interleukin-6, interleukin-1*β*, monocyte chemotactic protein 1, myeloperoxidase, cyclooxygenase-2, and inducible nitric oxide synthase. Administration of MaR 1 inhibited activation of nuclear factor kappa B (NF-*κ*b) and mitogen-activated protein kinases (MAPKs) in the liver of CCl4 treated mice. In conclusion, these results suggested the antioxidative, anti-inflammatory properties of MaR 1 in CCl4 induced liver injury. The possible mechanism is partly implicated in its abilities to inhibit ROS generation and activation of NF-*κ*b and MAPK pathway.

## 1. Introduction

Hepatitis is one of the most common liver diseases. Previous reports indicate that acute or chronic inflammation caused by various pathogenic factors such as viruses, bacteria, parasites, chemicals, drugs, alcohol, and other hepatotoxic agents is main cause of severe hepatocyte damage [1–6]. Carbon tetrachloride is well-known chemicals that can induce hepatotoxicity in humans and experimental animals by producing trichloromethyl radical (CCl3^•^) and trichloromethyl peroxy radical which can initiate lipid peroxidation. Lipid peroxidation occurs via reactive oxygen species (ROS) including superoxide radical (O_2_^−^) and hydroxyl radical (OH^−^). Both lipid peroxidation and ROS can induce cell death and cell destruction, while lipid peroxidation increases with development of acute liver injury in CCl4 treated rats [7]. Also, CCl4 can promote production of inflammatory cytokines and recruitment of inflammatory cells and cause liver dysfunction and damage [8]. Hepatic inflammation is considered as the hallmark of liver injury and early fibrosis, which is also found in extensive fibrosis, cirrhosis, and even cancer [9]. Therefore, inhibition of oxidative stress and inhibition of inflammation are two targets of alleviating CCL4 induced liver injury.

The proresolving mediators derived from polyunsaturated fatty acid are playing an important role in controlling inflammation and oxidative stress, including lipoxins, resolvins, and protectins [10, 11]. Resolvins and protectins can be biosynthesized from Omega-3 fatty acid eicosapentaenoic acid (EPA) and docosahexaenoic acid (DHA) and cause resolution of inflammation. In addition, DHA is the biosynthetic precursor of a new family of the most recently identified macrophage-derived proresolving mediators, termed maresins [12]. These new DHA-derived mediators are biosynthesized via 12-lipoxygenase in macrophages to produce 14S-hydroperoxydocosa-4Z, 7Z, 10Z, 12E, 16Z, 19Z-hexaenoic acid. This intermediate undergoes further conversion via 13(14)-epoxidation, which is quite crucial process for generation of 7R, 14S-dihydroxydocosa-4Z, 8E, 10E, 12Z, 16Z, 19Z-hexaenoic acid, termed as maresin 1 [12]. Recently, accumulating evidences both in vitro and in vivo indicate that MaR 1 can promote inflammation resolution and exert potent protective effects comparable to those reported for resolvins and lipoxins. In addition, a few reports demonstrated lipoxin A4 and its analogue, BML-111, could attenuate liver damage and inflammation response and prevent liver fibrosis [13–15]. Also, MaR 1 has displayed anti-inflammation and protective effects in murine model of colitis [16]. However, so far it is not yet known whether MaR 1 has protective effects in CCl4 induced liver injury. In this context, we investigated the impact of MaR 1 on liver injury in CCl4 treated mice and explored the possible mechanisms involved in this process. Our data revealed that MaR 1 can exert potential protective effects in CCl4 induced liver injury by inhibiting production of inflammatory mediators and reducing ROS production and lipid peroxidation. To obtain a better understanding of the underlying mechanisms, we investigated effects of MaR 1 on NF-*κ*b and MAPKs signal pathway in CCl4 induced liver injury.

## 2. Materials and Methods

### 2.1. Animals

All adult male BALB/c mice were purchased from the Animal Experimental Center of Wuhan University, aged 10 weeks with weights ranging from 20 to 25 g. All animal experiments were approved by the Animal Care and Use Committee of Tongji Medical College of Huazhong University of Science and Technology. The mice were fed with a standard laboratory diet and water ab libitum and maintained in a controlled environment under a 12 h light-dark cycle.

### 2.2. Reagents

7R-MaR 1 (Ann Arbor, MI, USA) was purchased from Cayman Chemical. CCl4 and olive oil were purchased from Nanjing Chemical Reagent Co. (Nanjing, China). The detection kits used for determination of alanine transaminase (ALT), aspartate transaminase (AST), superoxide dismutase (SOD), catalase (CAT), glutathione peroxidase (GP-X), reduced glutathione (GSH), myeloperoxidase (MPO), and thiobarbituric acid reactive substances (TBARS) were purchased from the Nanjing Jiancheng Institute of Biotechnology (Nanjing, China). 2′,7′-Dichlorofluorescein (DCF) and 2′,7′-dichlorofluorescein diacetate (DCFH-DA) were obtained from Sigma-Aldrich (St. Louis, MO, USA). TRIzol, PrimeScript RT Master Mix, and SYBR Green Master Mix were purchased from Takara (Japan). Mouse interleukin-6, interleukin-10, interleukin-1*β*, MCP-1, and TNF-*α* enzyme-linked immunosorbent assay (ELISA) kits were obtained from Dakewe Bioengineering Co., Ltd. (Shenzhen, China). Rabbit mAbs against ERK1/2, p38, JNK, phospho-p44/42 MAPK (Erk1/2), phospho-p38, and phospho-JNK were purchased from Cell Signaling Technology (Danvers, Mass). Rabbit mAbs against NF-*κ*b p65, inhibitor of NF-*κ*B (I*κ*B-*α*), and *β*-actin were purchased from Santa Cruz Biotechnology. Lamin B1 antibody was obtained from Epitomics (Burlingame, CA). Fetal bovine serum and RPMI-1640 culture medium were purchased from Gibco Life Technologies (Carlsbad, CA, USA). All other chemicals used were of highest commercial grade.

### 2.3. Experimental Procedure

Hepatic injury was induced by injecting i.p. with CCl4 0.1 mL/kg (10 mL/kg body weight, v/v = 1 : 99 in olive oil). To evaluate suitable dosage of MaR 1, CCl4 induced liver injury mice were treated with different dose of MaR 1 (0.03, 0.3, and 1 *μ*g/animal i.p.) once half an hour after CCl4 injection. After appropriate doses of MaR 1 were chosen, to explore protective effects of maresin 1, animals were divided into four groups: (1) control group, given appropriate vehicle throughout entire experiment, (2) MaR 1 group that received MaR 1 0.3 *μ*g/animal (i.p.), (3) CCl4 group that received vehicle as control group and then was given CCl4 0.1 mL/kg (10 mL/kg body weight, v/v = 1 : 99 in olive oil), and (4) MaR 1 + CCl4 treatment group that received MaR 1 0.3 *μ*g/animal (i.p.). Half an hour before MaR 1 administration, mice were injected with CCl4 0.1 mL/kg (10 mL/kg body weight, v/v = 1 : 99 in olive oil). Twenty-four hours after CCl4 injection, blood samples were collected from orbit after anesthesia by sodium pentobarbital and the mice were killed by cervical luxation. The liver was extracted quickly and weighted and then liver was cut into two pieces. One half was fixed by 4% paraformaldehyde and used for histopathological analysis. Other halves were used for other assessments.

### 2.4. Histopathological Examination

Halves of liver were fixed in 4% paraformaldehyde for 24 h and then embedded in paraffin and sectioned for 5 *μ*m thickness. Histopathological alteration was observed after hematoxylin-eosin (H&E) staining under light microscope (Olympus IX71, Tokyo, Japan). Six random fields were assessed for necrosis by standard morphologic criteria (e.g., loss of architecture) and area percentage of necrosis was measured by Image J (National Institutes of Health, Bethesda, MD).

### 2.5. Determination of Liver Enzymes

Serum was acquired by centrifugation of blood samples 800 g 15 min. Serum AST and ALT activities were measured with detection kits according to the manufacturer's instruction in a microplate reader.

### 2.6. Measurement of TBARS

The level of lipid peroxidation was assessed by TBARS assay as previously described [17]. In brief, liver tissues were homogenized in 0.01 M phosphate buffer saline (PBS) to make 1 : 10 (w/v) homogenates. Then the homogenates were centrifuged at 12000 g (4°C) for 15 min to acquire supernatants to determination of the level of TBARS according to the manufacturer's instruction.

### 2.7. Measurement of ROS Generation in Tissue

ROS generation in liver was determined by using dichlorofluorescein diacetate (DCFH-DA). 2′,7′-Dichlorofluorescein diacetate is nonpolar compound which can be converted to polar derivative by intracellular esterases. It can react with ROS to produce dichlorofluorescein (DCF), a highly fluorescent compound. ROS level was determined as described before with minor modifications [18]. Briefly, liver homogenates were diluted to 5 mg/mL in ice-cold 0.01 M PBS. The 1 mL reaction mixture includes PBS, 0.2 mL homogenates (5 mg/mL), and 10 *μ*L DCFH-DA (5 *μ*M). After 40 minutes of incubation at room temperature, the fluorescent product DCF was measured by using a spectrofluorimeter with excitation at 484 nm and emission at 530 nm. DCFH-DA in the absence of homogenates was used as background fluorescent. ROS formation was quantified from a DCF-standard curve.

### 2.8. The Cell Culture and Treatment

The human hepatic carcinoma cell line (HepG2) was obtained from Typical Cell Culture Collection Committee of the Chinese Academy of Sciences Library. The cells were cultured in RPMI-1640 culture medium containing 10% FBS and maintained in humidified incubator at 37°C and 5% CO_2_-enriched atmosphere. The exponential growth phase cells were collected and planted into 6-well plates at 2 × 10^5^ cells/well to adherent overnight. Then the cells were stimulated with CCl4 at concentration of 0.5% (v/v). Half an hour after CCl4 treatment, MaR 1 or vehicle was added. Twenty-four hours after CCl4 simulation, the cells were collected for further measurement.

### 2.9. ROS Assay In Vitro

ROS generation in cells was evaluated using DCFH-DA as a fluorescent probe previously described with minor modification [16]. In brief, cells were incubated with 10 *μ*M DCFH-DA for 30 minutes. After washing three times with PBS, HepG2 cells were observed and images were captured using fluorescence microscope (Olympus IX71, Tokyo, Japan). In addition, cells were collected for flow cytometric analysis using a FACSCanto II flow cytometer (BD Biosciences, San Jose, CA, USA) in order to obtain comparable data. The data was analyzed using FCS express 3 (De Novo Software) and the mean fluorescence intensity (MFI) was used to quantify the ROS generation.

### 2.10. SOD, GSH, CAT, and GP-X Activities Assay

Liver tissues were homogenized in ice-cold 0.01 M PBS and then the homogenates were centrifuged at 11000 ×g (4°C) for 10 minutes to obtain supernatants which were collected for antioxidants measurement. Levels of SOD, CAT, GSH, and GP-X in HepG2 cells (or liver homogenates) were assessed using their respective assay kits according to manufacturer's instruction. The activities of these antioxidants were evaluated according to absorbance determined via a spectrophotometer.

### 2.11. Cytokine Quantification by ELISA and Liver MPO Activity

The levels of IL-6, IL-1*β*, TNF-*α*, IL-10, and MCP-1 in BALB/c serum were determined by using respective enzyme-linked immunosorbent assay kits according to manufacturer's instruction. The MPO activity was assessed using a MPO detection kit according to manufacturer's instruction, which was determined according to absorbance measured at 450 nm via a spectrophotometer.

### 2.12. Quantitative Real-Time PCR

Total mRNAs were extracted from liver tissues by TRIzol regent as determined by the supplier's protocol and then reversely transcribed to cDNAs using PrimeScript RT Master Mix according to manufacturer's instruction. To determine the mRNAs' level of each gene, the real-time PCR was given SYBR Green Master Mix in the StepOnePlus real-time PCR system (Applied Biosystems, USA) for 45 cycles consisting of denaturation at 95°C for 30 s, annealing at 60°C for 30 s, and extension at 73°C for 30 s. Reaction was duplicated for each sample. Glyceraldehyde-3-phosphate dehydrogenase (GAPDH) was used as an internal control. The expression levels of COX-2 and iNOS were calculated using the 2^−ΔΔCt^ method. The primer pairs of COX-2 and iNOS are listed as previously described [19]: iNOS, Forward: 5′-GCCCTGCTTTGTGCGAAGTG-3′, Reverse: 5′-AGCCCTTTGTGCTGGGAGTC-3′; COX-2, Forward: 5′-CCTGGTCTGATGATGTATGC-3′, Reverse: 5′-GTATGAGTCTGCTGGTTTGG-3′. The primer pairs of GAPDH, CAT, GP-X, and SOD are listed: GAPDH: Forward: 5′-GGCCTTCCGTGTTCCTACC-3′, Reverse: 5′-GCCCAAGATGCCCTTCAGT-3′; CAT: Forward: 5′-TTCATCCGTGTAACCCGCTC-3′, Reverse: 5′-TGATCTGTTGTGAAATCAGTGC-3′; GP-X: Forward: 5′-AATCTATATCCTGGGACCCTGT-3′, Reverse: 5′-CCTCTCCAGGTGCCATAACC-3′; SOD: Forward: 5′-CGAGACATGTACGCCAAGGT-3′, Reverse: 5′-GCTTCTTGCGCTCTGAGTG-3′.

### 2.13. Western Blot Analysis

Hepatic tissue samples were removed from BALB/c mice and then were homogenized manually with glass homogenizers. The tissues were processed in Radioimmunoprecipitation Assay (Beyotime Biotechnology Company, Jiangsu, China) and centrifuged at 12000 g for 15 min at 4°C. Supernatant protein concentrations were assessed using BCA Protein Assay Kit (Beyotime Biotechnology Company, Jiangsu, China). Cytoplasmic and nuclear fractionations were performed with NE-PER Nuclear and Cytoplasmic Extraction Reagents (Pierce Biotechnology) according to the producer's instruction. Proteins were separated in 10% polyacrylamide sodium dodecyl sulfate gels and then were transferred to polyvinylidene fluoride membrane. The membranes were blocked using 5% nonfat milk for 2 hours and then were probed with antibodies against ERK1/2 (1 : 500), p38 (1 : 500), JNK (1 : 500), phospho-Erk1/2 (1 : 500), phospho-p38 (1 : 500), phospho-JNK (1 : 500), NF-*κ*b (1 : 500), I*κ*B-*α* (1 : 500), *β*-actin (1 : 1000), and lamin B1 (1 : 500), for an overnight incubation. After that, the membranes were incubated with secondary antibodies conjugated with horseradish peroxidase (HRP) for 1 hour. After washing, the proteins were detected with BeyoECL Plus (Beyotime Biotechnology Company, Jiangsu, China) and images were captured with the UVP imaging system.

### 2.14. Statistical Analysis

All data are indicated as means ± SEM. Statistical analysis was performed by one-way analysis of variance (ANOVA) plus Student-Newman-Keuls post hoc analysis. Statistical analyses were performed using the SPSS software version 17.0 (SPSS Inc., Chicago, IL). *P* < 0.05 was considered statistically significant.

## 3. Results

### 3.1. MAR 1 Ameliorates CCl4 Induced Hepatic Pathology

**Figure 1:**
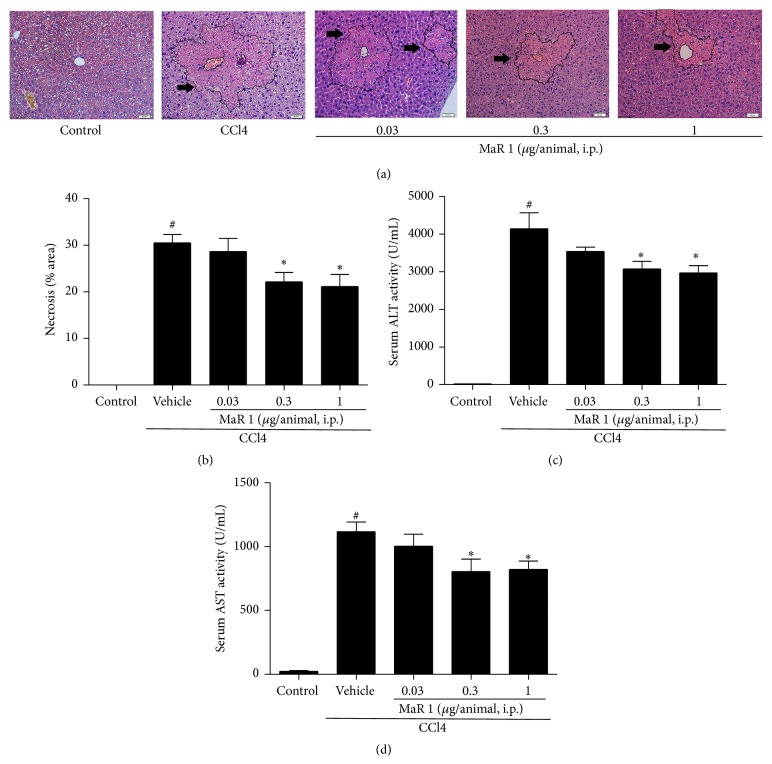
MaR 1 treatment inhibited CCl4 induced liver injury in mice. Representative micrographs and necrosis area from control mice (control), vehicle plus CCl4 (0.1 mL/kg i.p.), and MaR 1 treated of hematoxylin-eosin staining of liver section (a and b). The change of serum alanine aminotransferase (ALT) level (c) and aspartate aminotransferase (AST) activities (d) in CCl4 treated mice. Original magnification ×200. Arrows indicate necrotic area. Data are expressed as means ± SEM. *n* = 6. ^#^ *P* < 0.05 versus the control group, ^*∗*^ *P* < 0.05 versus the CCl4 treated group.

As shown in Figure 1(a), MaR 1 can significantly mitigate CCl4 induced hepatic injury. Intraperitoneal injection of MaR 1 induced histological changes. Control group showed normal liver tissue without massive cell necrosis and loss of hepatocyte architecture around the blood vessels. There were severe hepatocyte damage and necrosis at the centrilobular zones and influx of inflammatory cells 24 h after CCl4 administration. However the mice that received MaR 1 at 0.3 and 1 *μ*g/animal showed extensively alleviated CCl4 induced liver histopathological damage and necrosis while the 0.03 *μ*g/animal group displayed no significant protective effect (Figures 1(a) and 1(b)).

### 3.2. MaR 1 Decreases Serum AST and ALT Level

The serum ALT and AST activities are considered as two common biomarkers used to assess the liver damage. As shown in Figures 1(c) and 1(d), serum ALT and AST activities were significantly elevated (*P* < 0.05) 24 h after CCl4 administration compared to those in control group. Treatment with 0.03 *μ*g/animal MaR 1 did not show liver protective effect. However, treatment with 0.3 and 1 *μ*g/animal significantly (*P* < 0.05) decreased the activities of ALT (Figure 1(c)) and AST (Figure 1(d)) compared to those in CCl4 treated group, respectively.

### 3.3. MaR 1 Reduces ROS Level and the TBARS Content in Liver Tissue

**Figure 2:**
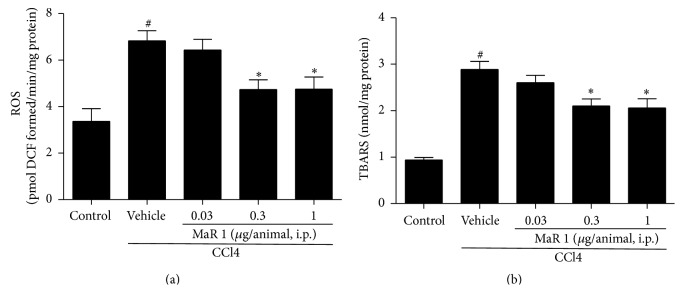
MaR 1 reduced the oxidative stress markers in CCl4 treated mice liver. Level of ROS (a) and TBARS (b). Each value was expressed as means ± SEM. *n* = 6. ^#^ *P* < 0.05 versus the control group, ^*∗*^ *P* < 0.05 versus the CCl4 treated group.

Increasing ROS level indicates production of free radicals, leading to oxidative stress, which is quite crucial in acute hepatic disorder. For this reason, we measured ROS in each group of mice using DCFH-DA. As shown in Figure 2(a), the CCl4 treated mice displayed a significant increase of ROS level compared with that in control mice. In contrast, treatment with MaR 1 at 0.3 and 1 *μ*g/animal markedly reduced ROS activities (*P* < 0.05) in liver tissue. However, MaR 1 (0.03 *μ*g/animal) had no significant effect in reducing ROS level in CCl4 treated animals.

It is well known that thiobarbituric acid reactive substances (TBARS) are formed as byproduct of lipid peroxidation. We determined hepatic TBARS level 24 h after CCl4 treatment. As shown in Figure 2(b), TBARS level significantly increased (*P* < 0.05) after CCl4 treatment compared to that in control animals. Notably, treatment of MaR 1 (0.3 and 1 *μ*g/animal) significantly reduced TBARS level (*P* < 0.05) compared with that in CCl4 treated group. As treatment with 0.3 *μ*g and 1 *μ*g/animal showed similar effect (Figures 1 and 2), we use the dose of 0.3 *μ*g/animal in subsequent animal experiments.

### 3.4. MaR 1 Inhibits CCl4 Induced ROS Generation In Vitro

**Figure 3:**
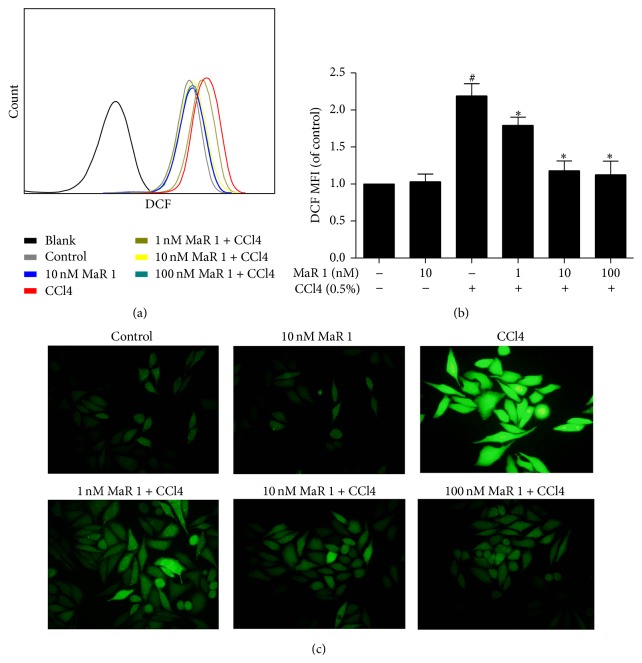
MaR 1 reduced CCl4 induced ROS generation in HepG2 cells. (a) Histograms of DCF green fluorescence. (b) Mean fluorescence intensity of fluorescent product of DCFH-DA of each group. Data are expressed as means ± SEM of three independent experiments. ^#^ *P* < 0.05 versus the control group, ^*∗*^ *P* < 0.05 versus the CCl4 treated group. (c) Cells were stained with DCFH-DA probe and images were captured using fluorescence microscope.

As ROS plays an important role in CCl4 induced damage, we explored ROS level in HepG2 cells using DCFH-DA. In fluorescence activated cell sorter analysis, we observed that CCl4 caused a significant ROS generation in HepG2 cells (Figures 3(a) and 3(b)) compared to ROS in control group. Notably, ROS generation was significantly inhibited with different concentration of MaR 1 (1, 10, and 100 nM) compared to that in CCl4 alone group (Figures 3(a) and 3(b)), and ROS level decreased in a dose-dependent manner. Furthermore, we got a similar result (Figure 3(c)) by using fluorescence microscopy.

### 3.5. MaR 1 Restores CCl4 Induced Antioxidants and GSH Production In Vitro

**Figure 4:**
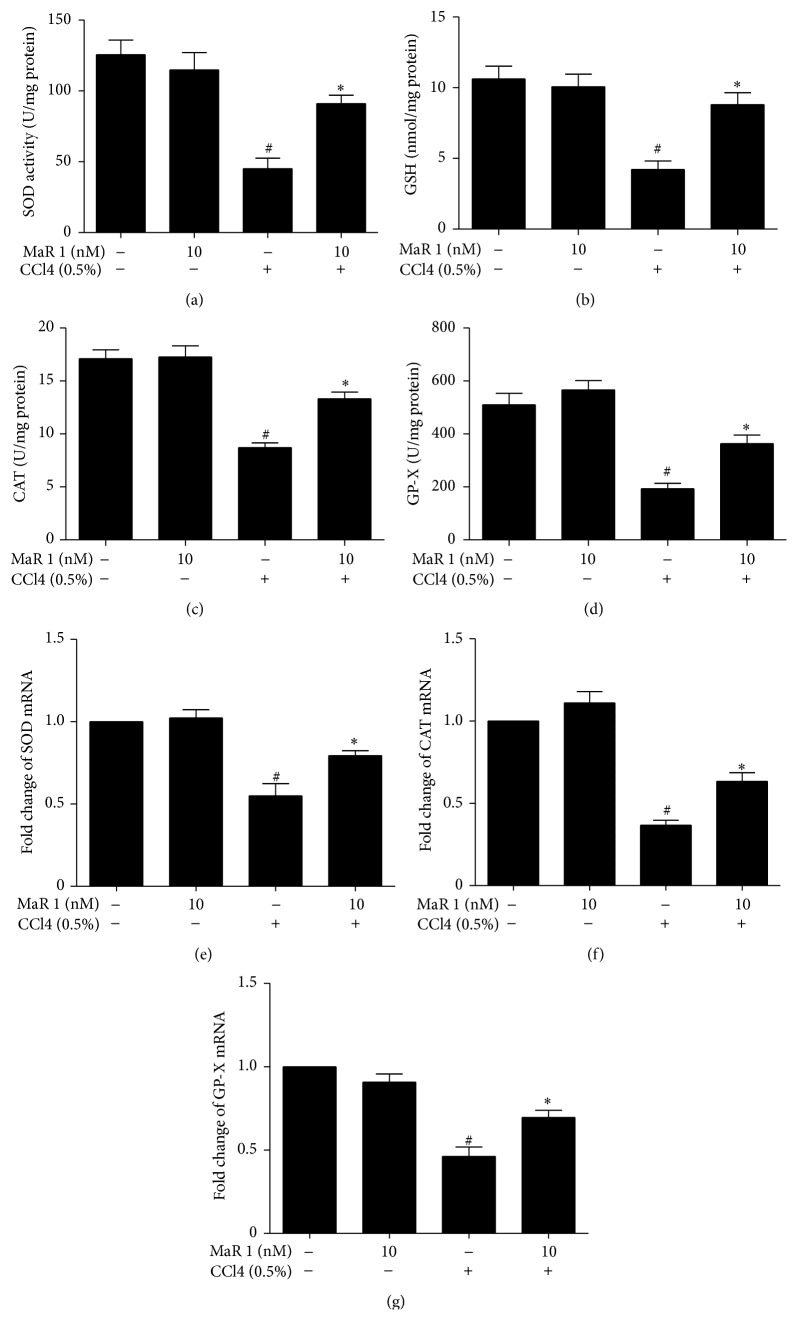
Treatment with MaR 1 restored antioxidant mediators in HepG2 cells. Effect of MaR 1 on T-SOD (a), GSH (b), CAT (c), and GP-X (d) level in CCl4-stimulated cells. Effect of MaR 1 on SOD (e), CAT (f), and GP-X (g) expression in CCl4 treated cells. Data are expressed as means ± SEM of three independent experiments. ^#^ *P* < 0.05 versus the control group, ^*∗*^ *P* < 0.05 versus the CCl4 treated group.

As treatment of 10 and 100 nM MaR 1 showed similar effect in vitro, the dose of 10 nM was chosen for measurement of intracellular enzymatic and nonenzymatic antioxidants. We found that CCl4 can decrease protein and transcription of antioxidative enzymes GP-X (Figures 4(d) and 4(g)), CAT (Figures 4(c) and 4(f)), and SOD (Figures 4(a) and 4(e)) and GSH level (Figure 4(b)). However treatment of MaR 1 can markedly elevate these antioxidants (Figure 4).

### 3.6. MaR 1 Restores CCl4 Induced Antioxidants and GSH Production in Liver

**Figure 5:**
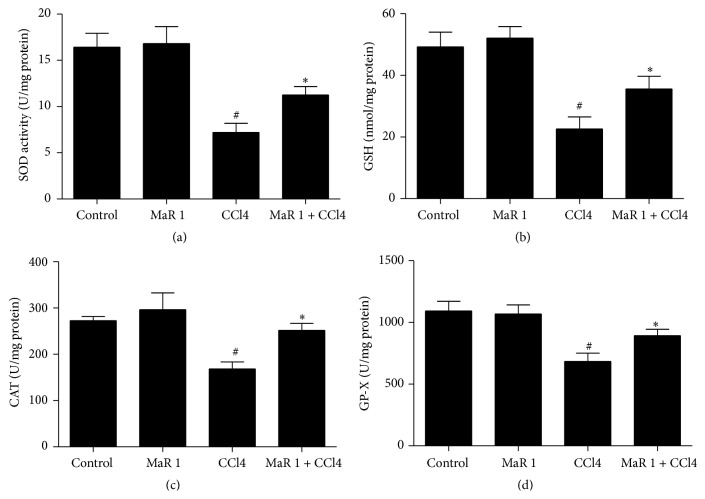
Administration with MaR 1 restored levels of antioxidant mediators in vivo. Effect of MaR 1 on liver T-SOD (a), GSH (b), CAT (c), and GP-X (d) level in CCl4-intoxicated mice. Data are expressed as means ± SEM. *n* = 6. ^#^ *P* < 0.05 versus the control group, ^*∗*^ *P* < 0.05 versus the CCl4 treated group.

To explore whether 0.3 *μ*g/animal MaR 1 can influence antioxidants level, we measured the activities of three antioxidative enzymes (GP-X, CAT, and SOD). We found that CCl4 administration resulted in significant decrease of hepatic SOD (Figure 5(a)), CAT (Figure 5(c)), and GP-X (Figure 5(d)) activities compared to those in control group. In contrast, treatment with MaR 1 greatly restored level of these antioxidative enzymes. Apart from that, glutathione (GSH) is an important endogenous antioxidant which mitigates damage caused by ROS. In our study, GSH level significantly decreased 24 h after CCl4 treatment (Figure 5(b)). However, administration of MaR 1 markedly increased GSH activity compared to that in CCl4 alone group. In addition, single treatment of MaR 1 had no effect on these antioxidants (Figures 5(a)–5(d)).

### 3.7. MaR 1 Reduces CCl4 Induced Proinflammatory Mediators and MPO Activity

**Figure 6:**
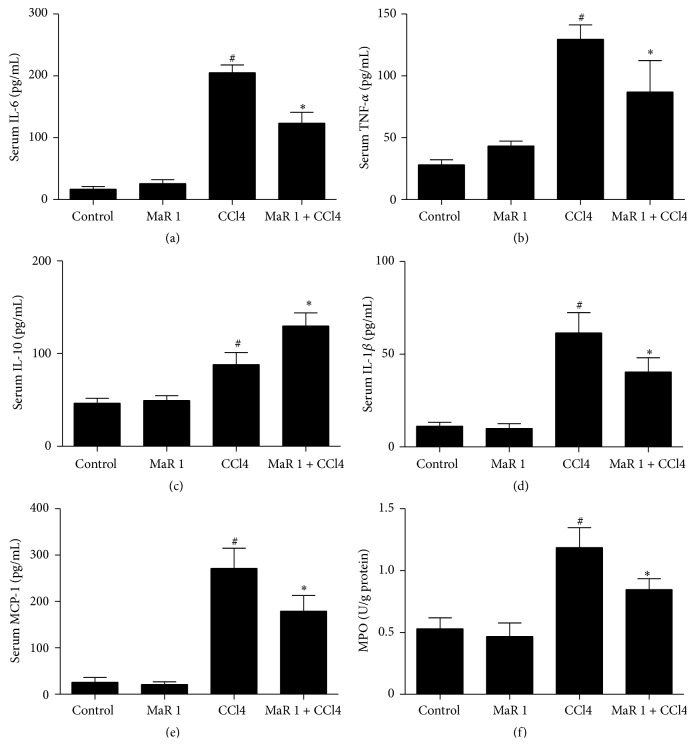
MaR 1 markedly inhibited production of IL-6, IL-1*β*, TNF-*α*, and MCP-1 in serum and MPO in liver tissue but increased IL-10 production in serum. Levels of IL-6 (a), TNF-*α* (b), IL-10 (c), IL-1*β* (d), and MCP-1 (e) in serum were measured by enzyme-linked immunosorbent assay. MPO activity (f) was measured in CCl4-intoxicated mice. Data are expressed as means ± SEM. *n* = 6. ^#^ *P* < 0.05 versus the control group, ^*∗*^ *P* < 0.05 versus the CCl4 treated group.

Cytokines were reported to play pivotal roles in hepatic injury and inflammatory response. In our study, single treatment with 0.3 *μ*g/animal MaR 1 had no influences on serum IL-6, IL-1*β*, TNF-*α*, IL-10, and MCP-1 (Figures 6(a)–6(e)). In CCl4 treated group, there were higher level proinflammatory cytokines such as IL-6, IL-1*β*, TNF-*α*, and MCP-1 (Figures 6(a), 6(b), 6(d), and 6(e)) compared to those in control group. The anti-inflammatory cytokine IL-10 also increased after MaR 1 treatment (Figure 6(c)). Treatment of 0.3 *μ*g/animal MaR 1 effectively reduced the levels of these proinflammatory cytokines in serum (Figures 6(a), 6(b), 6(d), and 6(e)). In addition, IL-10 slightly increased (Figure 6(c)) in CCl4 + MaR 1 group compared to that in CCl4 alone group (*P* < 0.05). We also evaluated MPO activity in liver homogenates. We found that MPO activity was significantly higher in CCl4 treated group (Figure 6(f)) compared to that in control group. Such upregulation was inhibited after treatment of 0.3 *μ*g/animal MaR 1. In addition, single administration had no influence on MPO activity.

### 3.8. MaR 1 Reduced Inflammatory Response

**Figure 7:**
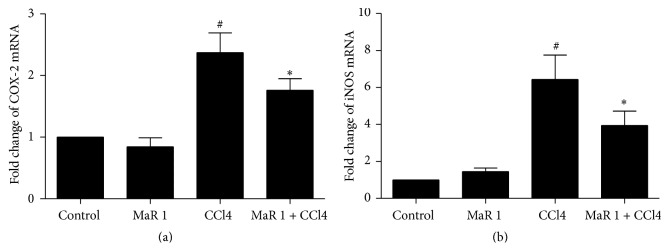
MaR 1 inhibited COX-2 and iNOS expression assessed by qRT-PCR. Effect of MaR 1 on liver COX-2 (a) and iNOS (b) expression in CCl4-intoxicated mice. Data are expressed as means ± SEM. *n* = 6. ^#^ *P* < 0.05 versus the control group, ^*∗*^ *P* < 0.05 versus the CCl4 treated group.

iNOS and COX-2 are two important inflammatory mediators implicated in inflammation [17, 20]. In our study, quantitative real-time PCR analysis indicated considerable upregulation of iNOS and COX-2 mRNA in liver tissue in CCl4 treated group compared to those in control group (Figures 7(a) and 7(b)). However, 0.3 *μ*g/animal MaR 1 administration significantly inhibited the expression of iNOS and COX-2 (*P* < 0.05) compared to CCl4 treated group (Figures 7(a) and 7(b)). In addition, treatment with MaR 1 alone had no influence (Figures 7(a) and 7(b)).

### 3.9. MaR 1 Inhibits CCl4 Induced MAPK Protein Phosphorylation

**Figure 8:**
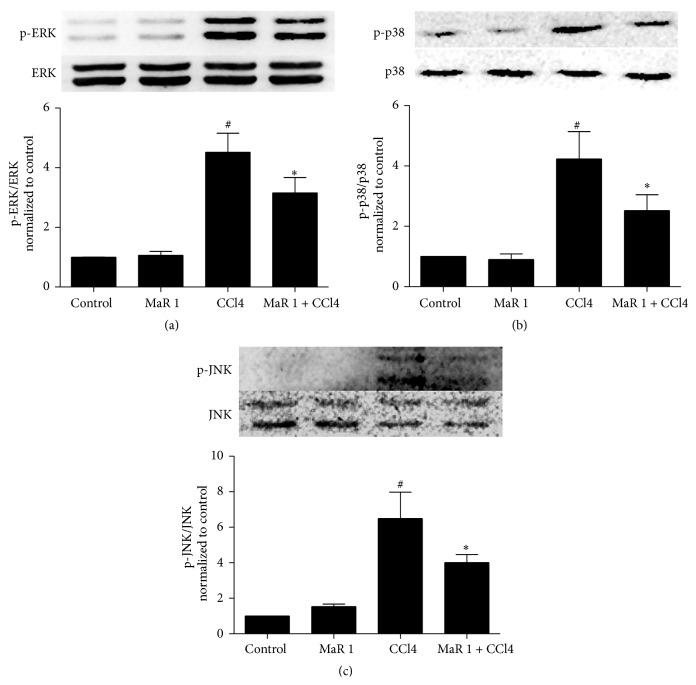
MaR 1 treatment inhibited mitogen-activated protein kinases activation. Total protein from whole cell lysate of liver tissue was extracted to analyze p-ERK/ERK (a), p-p38/p38 (b), and p-JNK/JNK (c). The control group is set as 1.0. The figures show representative results of six independent experiments. Data are expressed as means ± SEM. ^#^ *P* < 0.05 versus the control group, ^*∗*^ *P* < 0.05 versus the CCl4 treated group.

We examined phosphorylation of ERK, P38, and JNK in this study. Treatment of CCl4 resulted in significantly increased phosphorylation of ERK (Figure 8(a)), P38 (Figure 8(b)), and JNK (Figure 8(c)) compared to those in control group. Notably, treatment of 0.3 *μ*g/animal MaR 1 remarkably inhibited CCl4 induced phosphorylation of ERK, P38, and JNK in animals. Single treatment of MaR 1 showed no influence on MAPK phosphorylation.

### 3.10. MaR1 Suppresses NF-*κ*b p65 Nuclear Translocation and Rescues I*κ*B-*α* Degradation

**Figure 9:**
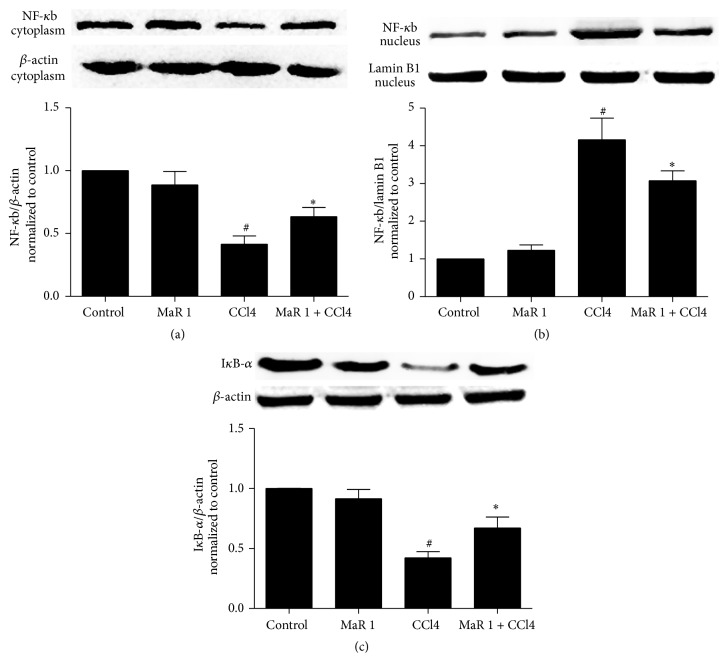
MaR 1 significantly inhibited NF-*κ*B p65 subunit translocation into the nucleus. (a) The cytoplasmic NF-*κ*B p65 subunit decreased in the CCl4 group but markedly increased with treatment of MaR 1. (b) Levels of nuclear NF-*κ*B p65 were promoted in the CCl4 group but significantly deceased with treatment of MaR 1. (c) Levels of I*κ*B-*α* in cytoplasm decreased after CCl4 administration but increased with treatment of MaR 1. A representative result from six independent experiments is shown. Quantification of cytoplasmic and nuclear NF-*κ*B p65 (a and b) bands from the experiments was normalized by *β*-actin or lamin B1. The control group is set as 1.0. Data are expressed as means ± SEM. ^#^ *P* < 0.05 versus the control group, ^*∗*^ *P* < 0.05 versus the CCl4 treated group.

NF-*κ*b pathway was tightly associated with CCl4 liver inflammation [21]. In present study, administration of CCl4 in mice greatly enhanced translocation of NF-*κ*b p65 from cytoplasm into nucleus (Figures 9(a) and 9(b)) compared to that in control group. In contrast, treatment of 0.3 *μ*g/animal MaR 1 significantly suppressed NF-*κ*b p65 translocation. The expression of I*κ*B-*α* significantly decreased in response to CCl4 injection. However, application of MaR 1 greatly inhibited I*κ*B-*α* degradation (Figure 9(c)). In addition, single treatment of MaR 1 had no influence on NF-*κ*b p65 nuclear translocation and I*κ*B-*α* degradation.

## 4. Discussion

CCl4 induces severe hepatic damage and oxidative stress resulting from CCl4 plays a pivotal role in this process. In our present study, administration of CCl4 significantly elicited hepatic damage, oxidative stress, and inflammatory response in BALB/c mice. However, treatment of MaR 1 can markedly reverse these above-mentioned changes. These indicated that MaR 1 mitigated CCl4 induced liver injury possibly through reducing oxidative stress and inflammation.

Liver is an important site for drug and toxicant metabolism. The metabolic process does no harm to liver in most cases while some toxic compounds can cause liver injury. ALT and AST in serum are widely used to assess hepatic function. The dramatic elevation of AST and ALT in serum denoted destruction of hepatic structure and damage of hepatocytes which caused elevation of cell membrane permeability and the release of ALT and AST into circulation. It has been well documented that CCl4 administration caused significant increase of ALT and AST in serum due to liver injury [7, 13, 17, 19]. In this study, we found treatment of MaR 1 partly reversed this effect and alleviated CCl4 induced liver dysfunction. Furthermore, MaR1 inhibited CCL4 induced histological damage by mitigating hepatocytes necrosis, the destruction of sinusoidal structure, and decreasing inflammatory cells infiltration.

Oxidative stress is critical in causing liver injury. CCl4 induced liver injury was characterized as increased oxidative stress and impairment of antioxidant defense. ROS is an important marker of oxidative stress and its generation contributed to the accumulation of lipid oxidation which leads to cell necrosis and liver injury [22]. The process of lipid peroxidation occurs when ROS oxidized membrane of cells, which can cause elevation of TBARS, eventually leading to change of cell structure and function. Reduction of oxidative stress has been implicated with significant role in alleviating hepatic damage in previous studies [19, 23]. In our study, the formation of ROS induced by CCl4 stimulation can be diminished by treatment of MaR 1. Both neutrophils and NADPH oxidase play important role in producing ROS. MPO level can reflect the degree of neutrophils infiltration [16]. In our study, we found maresin 1 could reduce MPO level in liver tissue after CCl4 administration (Figure 6(f)), which suggested reduction of neutrophils infiltration and subsequent ROS generation. Therefore, maresin 1 prevents the formation of ROS by CCl4 that may be associated with reduction of neutrophils infiltration. As ROS generation was inhibited, the level of TBARS was diminished after treatment with MaR 1, denoting that lipid peroxidation was also inhibited. Meanwhile, MaR1 restored antioxidative activity including GSH, GP-X, CAT, and SOD in vivo and in vitro (Figures 4 and 5). The loss of these mediators considerably caused free radicals accumulation and further liver damage [22]. It can be inferred that the protective effect of MaR1 on CCl4 induced liver injury is partly due to the modulation of oxidative and antioxidative balance.

Apart from oxidative stress, uncontrolled inflammation is another pathological cause implicated in CCl4 induced liver injury [22]. Proinflammatory cytokines such as IL-6, IL-1*β*, TNF-*α*, and MCP-1 and several enzymes are known to be crucial in inflammatory process and hepatic damage [15, 19, 24, 25]. It was previously found that increasing of MCP-1 and TNF may be associated with CCl4 induced liver injury and pretreatment of TNF antibody attenuated the liver injury induced by CCl4 [25]. Many studies also demonstrated that IL-1b and TNF-*α* play a key role in the development and maintenance of inflammatory and those cytokines' elevation is associated with many liver diseases [26, 27]. Proinflammatory TNF-*α* and IL-6 are major players in hepatic inflammation [28]. But it was found that IL-10 gene therapy attenuated CCl4 induced liver fibrosis in mice [29]. Therefore decreasing the level of those proinflammatory cytokines may be beneficial to liver and is mark of less inflammatory response. In the present study, MaR 1 significantly inhibited production of several proinflammatory cytokines including IL-6, IL-1*β*, TNF-*α*, and MCP-1 in serum. Meanwhile, MaR1 promoted the expression of anti-inflammatory cytokine IL-10. Furthermore, MaR1 inhibited iNOS and COX-2 expression in CCL4 treated livers. All these results suggest the protective effect of MaR 1 may be due to its anti-inflammatory ability.

We further investigated the possible mechanism and signaling pathway underlying such protective effect. The MAPK/NF-*κ*b pathway is important in inflammation. One type of stress that can activate MAPKs is oxidative stress induced by ROS. CCl4 induced ROS accumulation in mice liver and then ROS activated MAPKs pathway, which can further enhance the production of certain proinflammatory cytokines. In our study, administration of CCl4 activated MAPKs and treatment with MaR 1 significantly inhibited its activation in mice (Figure 8). MaR 1 can greatly inhibit ROS generation and oxidative stress, partly inhibiting phosphorylation of MAPKs and subsequently reducing inflammatory response. Therefore, MaR 1 may exert its protective effect by reducing ROS and subsequent MAPKs activation. NF-*κ*b is nuclear transcription factors which can regulate inflammation, proliferation, and apoptosis. In liver, it can be activated by cytokines and ROS [8]. Its activation is critical for maximal expression of many inflammatory cytokines (e.g., IL-1*β*, MCP-1, and TNF-*α*) which plays pivotal roles in liver injury. CCl4 can activate many signal pathways (such as MAPKs) which might converge on NF-*κ*b activation [8]. ROS induced by CCl4 in liver also can act as a messenger to NF-*κ*b [8]. The activation of NF-*κ*b nuclear translocation can increase the expression of proinflammatory molecules such as IL-1*β*, TNF-*α*, COX-2, and iNOS and lead to liver injury [21, 22, 30]. Our results showed that nuclear translocation of NF-*κ*b and phosphorylation of MAPKs both were inhibited with treatment with MaR 1 in CCl4 induced hepatic injury. Together, these studies suggested that protective effect of MaR 1 might be associated with suppression of ROS and inactivation of MAPK/NF-*κ*B signaling pathways.

## 5. Conclusions

Our study suggested for the first time that MaR 1 can protect liver by reducing oxidative stress and has potent protective effect against hepatic injury induced by inflammation through deceasing inflammatory mediators releasing. The possible mechanism, at least in part, included reduction of ROS generation through inhibiting CCl4 induced neutrophils infiltration, subsequently suppressed ROS induced activation of MAPKs and NF-*κ*B. For one thing, reduction of ROS denoted relief of oxidative stress in liver. For another, inhibition of ROS induced MAPKs and NF-*κ*B activation can reduce proinflammatory response which may be harmful in liver injury. Moreover, we demonstrated MaR 1 mitigated CCl4 induced liver dysfunction and histopathologic change. Taken together, administration of MaR 1 may be a new potential therapeutic approach in treatment of liver injury induced by toxic substance.

## Abbreviations

ALT: Alanine transaminase
AST: Aspartate transaminase
CAT: Catalase
CCl4: Carbon tetrachloride
COX-2: Cyclooxygenase-2
DCF: Dichlorofluorescein
DCFH-DA: Dichlorofluorescein diacetate
DHA: Docosahexaenoic acid
ELISA: Enzyme-linked immunosorbent assay
GP-X: Glutathione peroxidase
GSH: Glutathione
IL: Interleukin
iNOS: Nitric oxide synthase
MaR 1: Maresin 1
MAPKs: Mitogen-activated protein kinases
MCP-1: Monocyte chemotactic protein 1
MPO: Myeloperoxidase
ROS: Reactive oxygen species
SOD: Superoxide dismutase
TBARS: Thiobarbituric acid reactive substances.

## References

[1] Kennedy E. M., Bassit L. C., Mueller H. (2014). Suppression of hepatitis B virus DNA accumulation in chronically infected cells using a bacterial CRISPR/Cas RNA-guided DNA endonuclease. *Virology*.

[2] Markwick L. J., Riva A., Ryan J. M. (2015). Blockade of PD1 and TIM3 restores innate and adaptive immunity in patients with acute alcoholic hepatitis. *Gastroenterology*.

[3] Ferrari C. (2015). HBV and the immune response. *Liver International*.

[4] Chen X., Yang X., Li Y. (2014). Follicular helper T cells promote liver pathology in mice during *Schistosoma japonicum* infection. *PLoS Pathogens*.

[5] Marques P. E., Oliveira A. G., Pereira R. V. (2015). Hepatic DNA deposition drives drug-induced liver injury and inflammation in mice. *Hepatology*.

[6] Wang J.-B., Pu S.-B., Sun Y. (2014). Metabolomic profiling of autoimmune hepatitis: the diagnostic utility of nuclear magnetic resonance spectroscopy. *Journal of Proteome Research*.

[7] Campo G. M., Avenoso A., Campo S. (2004). Hyaluronic acid and chondroitin-4-sulphate treatment reduces damage in carbon tetrachloride-induced acute rat liver injury. *Life Sciences*.

[8] Ma J.-Q., Ding J., Zhang L., Liu C.-M. (2014). Ursolic acid protects mouse liver against CCl4-induced oxidative stress and inflammation by the MAPK/NF-*κ*B pathway. *Environmental Toxicology and Pharmacology*.

[9] Bishayee A. (2014). The role of inflammation and liver cancer. *Advances in Experimental Medicine and Biology*.

[10] Serhan C. N., Hong S., Gronert K. (2002). Resolvins: a family of bioactive products of omega-3 fatty acid transformation circuits initiated by aspirin treatment that counter proinflammation signals. *The Journal of Experimental Medicine*.

[11] Yaxin W., Shanglong Y., Huaqing S. (2014). Resolvin D1 attenuates lipopolysaccharide induced acute lung injury through CXCL-12/CXCR4 pathway. *Journal of Surgical Research*.

[12] Serhan C. N., Yang R., Martinod K. (2009). Maresins: novel macrophage mediators with potent antiinflammatory and proresolving actions. *The Journal of Experimental Medicine*.

[13] Zhang L., Wan J., Li H. (2007). Protective effects of BML-111, a lipoxin A_4_ receptor agonist, on carbon tetrachloride-induced liver injury in mice. *Hepatology Research*.

[14] Xia J., Zhou X.-L., Zhao Y., Zhu Y.-Q., Jiang S., Ni S.-Z. (2013). Roles of lipoxin A4 in preventing paracetamol-induced acute hepatic injury in a rabbit model. *Inflammation*.

[15] Zhou X.-Y., Yu Z.-J., Yan D. (2013). BML-11, A lipoxin receptor agonist, protected carbon tetrachloride-induced hepatic fibrosis in rats. *Inflammation*.

[16] Marcon R., Bento A. F., Dutra R. C., Bicca M. A., Leite D. F. P., Calixto J. B. (2013). Maresin 1, a proresolving lipid mediator derived from omega-3 polyunsaturated fatty acids, exerts protective actions in murine models of colitis. *Journal of Immunology*.

[17] Domitrović R., Jakovac H., Blagojević G. (2011). Hepatoprotective activity of berberine is mediated by inhibition of TNF-*α*, COX-2, and iNOS expression in CCl_4_-intoxicated mice. *Toxicology*.

[18] Shinomol G. K., Muralidhara (2007). Differential induction of oxidative impairments in brain regions of male mice following subchronic consumption of Khesari dhal (*Lathyrus sativus*) and detoxified Khesari dhal. *NeuroToxicology*.

[19] Zhang H., Yu C.-H., Jiang Y.-P. (2012). Protective effects of polydatin from *Polygonum cuspidatum* against carbon tetrachloride-induced liver injury in mice. *PLoS ONE*.

[20] Tipoe G. L., Leung T. M., Liong E. (2006). Inhibitors of inducible nitric oxide (NO) synthase are more effective than an NO donor in reducing carbon-tetrachloride induced acute liver injury. *Histology and Histopathology*.

[21] Muriel P. (2009). NF-*κ*B in liver diseases: a target for drug therapy. *Journal of Applied Toxicology*.

[22] Hu L., Li L., Xu D. (2014). Protective effects of neohesperidin dihydrochalcone against carbon tetrachloride-induced oxidative damage in vivo and in vitro. *Chemico-Biological Interactions*.

[23] Zhang F., Wang X., Qiu X. (2014). The protective effect of esculentoside a on experimental acute liver injury in mice. *PLoS ONE*.

[24] Río A., Gassull M. A., Aldeguer X., Ojanguren I., Cabré E., Fernández E. (2008). Reduced liver injury in the interleukin-6 knockout mice by chronic carbon tetrachloride administration. *European Journal of Clinical Investigation*.

[25] Sato A., Nakashima H., Nakashima M. (2014). Involvement of the TNF and FasL produced by CD11b kupffer cells/macrophages in CCl_4_-induced acute hepatic injury. *PLoS ONE*.

[26] Shin D.-S., Kim K. W., Chung H. Y., Yoon S., Moon J.-O. (2013). Effect of sinapic acid against carbon tetrachloride-induced acute hepatic injury in rats. *Archives of Pharmacal Research*.

[27] Weber L. W. D., Boll M., Stampfl A. (2003). Hepatotoxicity and mechanism of action of haloalkanes: carbon tetrachloride as a toxicological model. *Critical Reviews in Toxicology*.

[28] Fu Y., Zheng S., Lin J., Ryerse J., Chen A. (2008). Curcumin protects the rat liver from CCl4-caused injury and fibrogenesis by attenuating oxidative stress and suppressing inflammation. *Molecular Pharmacology*.

[29] Chou W.-Y., Lu C.-N., Lee T.-H. (2006). Electroporative interleukin-10 gene transfer ameliorates carbon tetrachloride-induced murine liver fibrosis by MMP and TIMP modulation. *Acta Pharmacologica Sinica*.

[30] Wang H., Wang L., Li N. L. (2014). Subanesthetic isoflurane reduces zymosan-induced inflammation in murine Kupffer cells by inhibiting ROS-activated p38 MAPK/NF-*κ*B signaling. *Oxidative Medicine and Cellular Longevity*.

